# Comparison of Immune Response Assessment in Colon Cancer by Immunoscore (Automated Digital Pathology) and Pathologist Visual Scoring

**DOI:** 10.3390/cancers14051170

**Published:** 2022-02-24

**Authors:** Isabelle Boquet, Alboukadel Kassambara, Alfred Lui, Alicia Tanner, Marie Latil, Yoann Lovera, Fanny Arnoux, Fabienne Hermitte, Jérôme Galon, Aurelie Catteau

**Affiliations:** 1 Veracyte, 13288 Marseille, France; isabelle.boquet@haliodx.com (I.B.); alboukadel.kassambara@haliodx.com (A.K.); alicia.tanner@haliodx.com (A.T.); marie.latil@haliodx.com (M.L.); yoann.lovera@haliodx.com (Y.L.); fanny.arnoux@haliodx.com (F.A.); fabienne.hermitte@haliodx.com (F.H.); jerome.galon@haliodx.com (J.G.); 2 Innovative Pathology Medical Group, Torrance, CA 90503, USA; aluibdl@gmail.com; 3 INSERM (Institut National de la Santé et de la Recherche Médicale), Laboratory of Integrative Cancer Immunology, 75006 Paris, France; 4 Equipe Labellisée Ligue Contre le Cancer, 75006 Paris, France; 5 Centre de Recherche des Cordeliers, Sorbonne Université, Université de Paris, 75006 Paris, France

**Keywords:** colon cancer, low-risk, high-risk, digital pathology, Immunoscore^®^, T-score, recurrence, misclassification, quantification, pathologist, stratification

## Abstract

**Simple Summary:**

The immune response to colon cancer (CC) is highly variable among patients and is clinically relevant. In this study, we compared the immune response assessment for early-stage CC, as measured by Immunoscore (IS), to pathologist visual scoring of the CD3+ and CD8+ T-cell densities at the tumor site (T-score). The objectives were to determine the inter-observer agreement between pathologists and the concordance between the two methods. Agreement between pathologists was minimal to weak. Moreover, a weak concordance between the two methods was observed, leading to misclassification of 48% of cases by pathologist scoring. Due to the high level of immune infiltrate heterogeneity resulting in disagreement of interpretation among pathologists, IS is unlikely to be reproduced via non-standardized methods.

**Abstract:**

Adjunction of immune response into the TNM classification system improves the prediction of colon cancer (CC) prognosis. However, immune response measurements have not been used as robust biomarkers of pathology in clinical practice until the introduction of Immunoscore (IS), a standardized assay based on automated artificial intelligence assisted digital pathology. The strong prognostic impact of the immune response, as assessed by IS, has been widely validated and IS can help to refine treatment decision making in early CC. In this study, we compared pathologist visual scoring to IS. Four pathologists evaluated tumor specimens from 50 early-stage CC patients and classified the CD3+ and CD8+ T-cell densities at the tumor site (T-score) into 2 (High/Low) categories. Individual and overall pathologist scoring of immune response (before and after training for immune response assessment) were compared to the reference IS (High/Low). Pathologists’ disagreement with the reference IS was observed in almost half of the cases (48%) and training only slightly improved the accuracy of pathologists’ classification. Agreement among pathologists was minimal with a Kappa of 0.34 and 0.57 before and after training, respectively. The standardized IS assay outperformed expert pathologist assessment in the clinical setting.

## 1. Introduction 

The important role of immune response to the tumor has been demonstrated in numerous solid cancers [1,2,3,4,5,6,7], including Colon Cancer (CC) [8,9,10,11,12,13,14,15,16], with a high-level of tumor-infiltrating lymphocytes (TILs) being consistently associated with a favorable prognosis. Various methods with different cutoff values have been used to assess immune cell infiltration. Hematoxylin and eosin (H&E) staining of tumor tissue is the most frequently used histochemical stain in clinical and research laboratories. However, with this method, it is difficult to count the number of TILs in cancer cell nests [2]. The reproducibility of the immune response evaluation by visual examination of H&E slides was previously reported and showed a low level of concordance between the 11 expert observers (4% of 268 cases evaluated) [2]. Due to heterogeneity of TILs and the subjectivity of its evaluation on H&E slides by pathologists, such a method was not reliable enough for a therapeutic decision-making process.

Therefore, markers with more accuracy and added clinical value are needed. Moreover, consensus recommendations for scoring TILs for diagnostic purposes, translational research, and the clinical trials are required. The integration of the IS assay into pathology clinical practice can help to ensure the higher level of accuracy and efficiency for characterization of immune response [17,18,19,20].

We previously showed that of all immune cells involved in the in situ immune reaction, CD3+ and CD8+ T-lymphocyte cells (specific populations of tumor-infiltrating lymphocytes; TILs) provided the optimal combination for prognostic purpose. The accuracy of prediction of survival times for the different patient groups was greater with a combined analysis of the center of tumor (CT) and the invasive margin (IM) regions than with a single-region analysis [21]. CD3 and CD8 were also chosen as markers because of the quality of the staining and the stability of these antigens. We then developed and validated the immune-based international consensus IS assay [2]. Immunoscore^®^ values are reported based on predefined cutoffs and given one of five category scores (IS 0 to IS 4) that are combined into two relevant clinical risk categories: IS Low (IS 0–1) and IS High (IS 2–4). These distinguish tumors with low versus high immune infiltration that are associated with high versus low risk of recurrence, respectively. IS is now recommended for use in conjunction with the TNM classification system to estimate prognosis for early-stage CC patients in the ESMO Clinical Practice Guidelines [22,23]. In a large international study of more than 3500 stage I-III CC patients, in high-risk stage II patients, IS identified a large fraction of patients (70%) whose risk for recurrence was similar to that of low-risk stage II patients when not treated with chemotherapy [3,23,24]. This strongly suggests a clinical utility for the IS assay to identify patients having a low biological recurrence risk despite the presence of pathologic high-risk features that might otherwise trigger adjuvant chemotherapy. These patients may avoid unnecessary treatment and its attendant toxicities. In addition, IS was shown to be a powerful prognostic marker for stage III CC patients in two randomized phase III clinical trials [3,4] and also predicted response of adjuvant chemotherapy in two independent cohorts [4,6].

The analytical validation of IS has been demonstrated previously [20,25]. Immunoscore^®^ was deemed to be a robust, reproducible, quantitative, and standardized immune assay, with a high prognostic performance, independent of all of the prognostic markers currently used in clinical practice. The immune response was introduced for the first time into the latest (5th) edition of the WHO Digestive System Tumors as “an essential and desirable diagnostic criteria for CC”. Furthermore, the 2020 ESMO Clinical Practice Guidelines for CC included IS to refine the prognosis, stratify patients according to risk, and thus adjust the chemotherapy decision-making process, although its role in predicting an adjuvant chemotherapy effect is uncertain. Therefore, it is important to compare the performance of the standardized consensus digital pathology IS to an evaluation of the immune response by visual examination of H&E slides or by a visual examination of CD3+- and CD8+-stained slides by expert pathologists.

Here, we compared the performance of automated digital pathology (using IS) and pathologist visual scoring of CD3+ and CD8+ T-cell densities at the tumor site (T-score) for assessment of immune response in patients with CC. The performance of each of the two methods in assessing the immune response status and the impact of misclassifications of the risk of recurrence on patient management and treatment decisions was evaluated.

## 2. Material and Methods

This study compared the immune response assessment in early-stage CC by two methods: (1) expert pathologist evaluation of CD3+ and CD8+ stained slides at the tumor site (T-score) in two steps: (i) without training and (ii) with training and (2) artificial intelligence assisted digital pathology (IS).

### 2.1. Case Selection

Representative high-resolution scanned images of CD3+ and CD8+ single-stained tumor specimens from 50 patients were selected from the IDEA-France study [4]. The mean densities of CD3+ and CD8+ T-cells quantified in the CT and IM were converted into IS with predefined cutoffs [26,27]. Immunoscore® uses standardized percentile values (0–100%), and the algorithm categorizes the continuous Immunoscore® into five groups (0, 1, 2, 3, and 4). A predefined two-level classification (2 groups of recurrence risk) uses predefined cutoffs corresponding to IS-Low with a mean percentile of 0–25% (IS 0–1) and IS-High with a mean percentile of >25–100% (IS 2–4), consistent with the validated assay cutoffs determined in the Society for Immunotherapy of Cancer (SITC) study [6], with IS-Low indicating a poor prognosis (high-risk of relapse) and IS-High is indicative of a good prognosis.

The mean of the 4 percentiles (mP) obtained for CD3+ and CD8+, either in the CT or IM, was calculated for each specimen collected from 50 patients and grouped into 10 categories (0–10%, 10–20%, 20–30%, 30–40%, 40–50%, 50–60%, 60–70%, 70–80%, 80–90%, and 90–100%). Within each category, 5 cases were randomly selected to ensure a uniform distribution of 50 cases along the dynamic range of IS at the level of mP. Then IS was categorized into two groups.

The subset of 20 cases, for which IS was around a 25% mP clinical cutoff point, were analyzed separately. This subgroup consisted of 10 cases with IS-Low (≤25%) and 10 cases with IS-High.

### 2.2. Pathologist Visual Assessment

Four expert pathologists with broad experience in gastrointestinal cancer pathology independently assessed the immune infiltration (CD3+ and CD8+ T-cells) for the 50 selected cases through qualitative visual and manual scoring via an online secured-access web gallery. Pathologists were asked to classify each marker density and to sort them into three categories (Low, Intermediate, and High) and a final 2-class T-score (Low or High) was generated in accordance with clinical reporting. The images were analyzed blindly without training instructions. To avoid a learning bias, cases were analyzed by each pathologist in a pre-specified, individualized, and randomized order.

### 2.3. Pathologist Training

In a second step, the pathologists were trained to assess densities of each marker at the 25% mP (separating Low and Intermediate staining) across a selection of four illustrative images (Table 1). In order to recognize heterogeneity in T-cell infiltrates from different regions in multiple tumors but yielding equivalent T-scores, pathologists were further provided a set of 12 images that represented a spectrum of CD3+ and CD8+ densities across the CT and IM regions (Table 1).

The four pathologists repeated immune infiltration evaluation on the same 50 selected cases after this training and reported their classification category for CD3+ and CD8+ T-cells in both the CT and IM and the overall category (Low/Intermediate/High) for each case. A final 2-class T-score (Low or High) was generated in accordance with clinical reporting of the Immunoscore^®^. The immune infiltration assessment data were captured using a data collection Excel spreadsheet and analyzed.

### 2.4. Repeatability Evaluation of IS

The 50 reference cases were internally analyzed three times to evaluate repeatability of the IS method. The IS module (Immunoscore^®^ Analyzer, Veracyte, Marseille, France) was used for automatic detection of the CT and IM, quantification of CD3+- and CD8+-stained T-cells, and classification of the reference cases into the clinical IS categories. Each IS repetition (identified as DP1, DP2, and DP3) and validation of the results were carried out by two histotechnicians who evaluated the technical parameters, including immunoperoxidase staining quality (the histotechnicians are experienced and expert in performing quality control analysis of IS cases). The IS results and the name of the histotechnician were captured using a data collection Excel spreadsheet.

### 2.5. Statistical Analysis

The statistical analysis was used to explore the following types of concordance: between individual pathologist assessment and IS for all cases (*n* = 50) and for the subset of cases around the clinical 25% IS cutoff (*n* = 20) before and after training, inter-pathologists’ agreement with visual assessment of T-score, and among three repeated IS assessments.

### 2.6. Agreement Evaluation

The Cohen’s Kappa coefficient was used to evaluate agreement of Immunoscore® results between the two rating methods, IS and pathologists’ scoring. The Fleiss’s Kappa coefficient test, an extension of the Cohen’s kappa, was used to compute the agreement between multiple observers’ assessments. In accordance with McHugh et al. [28], the level of agreement was categorized according to the Kappa values as none (0–20%), minimal (21–39%), weak (40–59%), moderate (60–79%), strong (80–90%), and almost perfect (>90%). A negative Kappa indicated that there was less agreement than would be expected by chance, given the marginal distributions of ratings.

## 3. Results

### 3.1. Comparison of Individual Pathologist Visual Assessment to Is before Training

**Figure 1:**
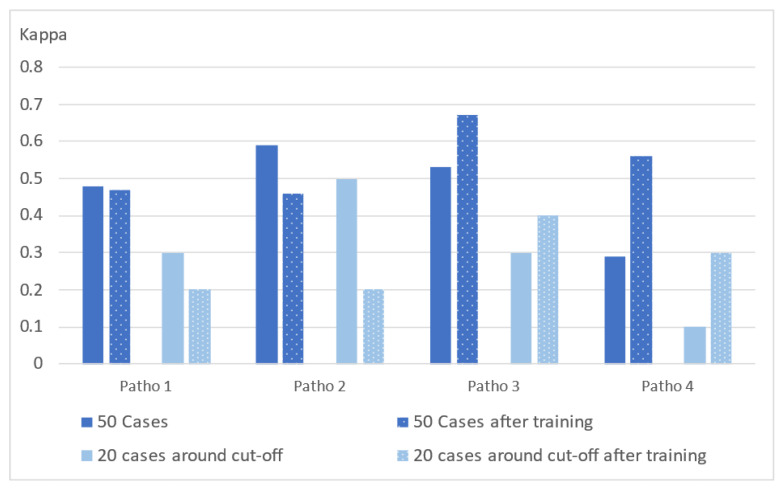
Bar plot showing agreement between individual pathologist visual assessment (T-score) and the reference Immunoscore^®^ (IS) before (plain bars) and after (dotted bars) training. The *y*-axis shows the level of agreement according to the Cohen’s kappa value: none (0–0.20), minimal (0.21–0.39), weak (0.40–0.59), moderate (0.60–0.79), strong (0.80–0.90), and almost perfect (0.91–1). For each pathologist (Patho 1–4), T-score was expressed as High or Low for 50 cases along the dynamic range of IS (dark blue) or 20 cases around the clinical cut-off of 25% (light blue).

Without previous training, the agreements were weak between pathologists’ T-score classification and the reference IS for the immune infiltration assessment of 50 CC cases (Figure 1, plain dark blue bars). The mean agreement (Cohen’s Kappa) for pathologists’ T-score classification compared to the reference IS was 0.47 (minimum and maximum agreements were (0.29–0.59)). The maximum agreement rate with the reference IS was 82% (Cohen’s Kappa of 0.59) for pathologist #2 and 80% for the three other pathologists #1, #3, and #4, with Cohen’s Kappa from 0.29 to 0.53 (Figure 1, plain dark blue bars). The lowest percentage of negative agreement between T-score and IS, 25%, was observed for pathologist #4, while the lowest positive percent agreement was observed for pathologists #2 and #3 (79%).

The disagreement rates for T-score classification versus the reference IS for each pathologist were even higher for the 20 cases with IS percentiles around the clinical cutoff. A minimal level of agreement was reached by pathologists’ visual evaluation compared to the reference IS: the mean agreement (Cohen’s Kappa) for pathologists T-score classification compared to the reference IS was 0.30 (minimum and maximum agreements were (0.10–0.50); Figure 1, plain light blue bars).

### 3.2. Comparison of Individual Pathologist Visual Assessment to Is after Training

After training, a moderate level of agreement between the pathologist T-score visual assessment and the reference IS on the 50 cases was reached for one pathologist (#3; Cohen’s Kappa of 0.67) while it remained weak for all other pathologists (Cohen’s Kappa ranging from 0.46 to 0.56). The mean agreement (Cohen’s Kappa) for pathologists’ T-score classification compared to the reference IS was 0.54 (minimum and maximum agreements were (0.46–0.67); Figure 1, dotted dark blue bars).

The best agreement rate for classification of the 20 cases around the clinical cutoff after training was observed for pathologist #3 (70%) with a corresponding weak Cohen’s Kappa agreement of 0.40 (versus 0.30 before training; Figure 1, dotted light blue bars).

**Figure 2:**
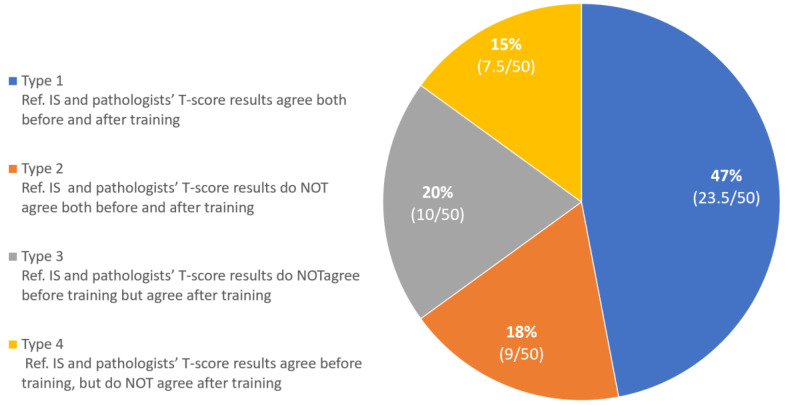
Figure **2.** Distribution of agreement types between T-score visual assessment and the reference IS (Low, Intermediate, High) in a set of 50 colon cancer cases, before and after training (average of cases falling in each type is reported in parentheses).

The impact of training was further assessed by evaluating the four different types of “agreement” (i.e., combining concordance or discordance before and after training, Figure 2). On average, training had a positive impact in 20% of the analyzed cases (Type 3). However, training had no impact for 18% of the cases (Type 2) and even worsened the concordance between the visual assessment and IS in 15% of the cases (Type 4).

### 3.3. Inter-Pathologist Agreement with Visual Assessment of T-Score

**Figure 3:**
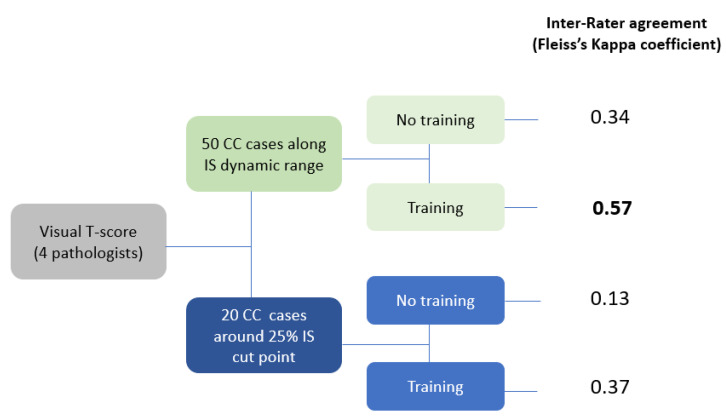
Fleiss’s Kappa values for inter-observer agreement for T-score classification (50 selected CC cases and 20 cases around the clinical IS cutoff point) before and after training.

The inter-observer agreement for the 50 selected CC cases into T-score classification was weak before training (Fleiss’s Kappa of 0.34) and was still weak after training (Fleiss Kappa of 0.57; Figure 3). The agreement rates were minimal or nonexistent for the 20 cases around the clinical IS cutoff point (Fleiss Kappa of 0.13 and 0.37, before and after training, respectively; Figure 3).

### 3.4. Disagreement of Pathologist Visual Assessment with the Reference IS

**Figure 4:**
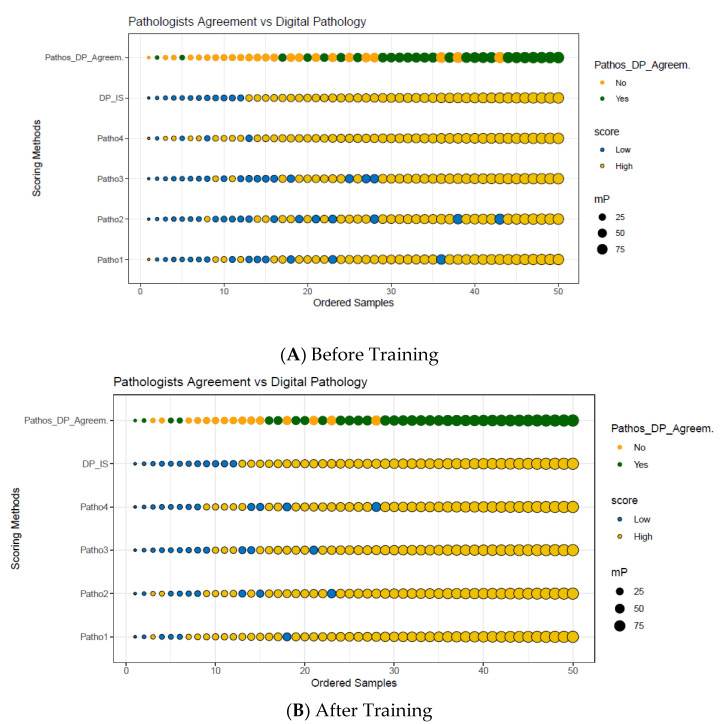
Graphical plot representing the agreement between each of the four pathologists visual T-score and IS before (**A**) and after training (**B**) of 50 colon cancer cases. Reference IS scores (Low and High) from 50 colon cancer cases (x axis) are plotted against the pathologist visual T-score and IS methods (y axis). Dark green circles indicate an agreement between all pathologists and the reference digital pathology IS method. Bright orange circles indicate disagreement between at least one of the pathologists and the reference IS. The mean percentiles (mP) of the CD3+ and CD8+ T-cells densities are represented as circles, whose size is proportional to the mP value observed for each case. The 50 cases were ranged from the lowest mP to the highest mP and IS was translated into 2-category classification (dashed line): Low IS (mP ≤ 25%) and High IS (mP ˃ 25%); the T-score classification for each pathologist is represented by blue circles with a black outline (Low T-scores) and yellow circles with a black outline (High T-scores). Abbreviations: Patho, pathologist; mP, mean percentile; DP, digital pathology.

Pathologist disagreement with the reference IS, as defined as the percentage of cases for which at least one pathologist assessment was not concordant with the reference IS, was observed in nearly half of the cases without training (48%; 24 out of 50; Figure 4A) and in 30% of the cases (15 out 50) after training (Figure 4B). The analysis of the 20 CC cases around the IS clinical cutoff resulted in even lower concordance with the overall disagreement rate as high as 80% and 65% for before and after training, respectively. Pathologists agreed only on three High T-scores and one Low T-score out of 20 cases before training (Figure 4A) and on 5 and 2 cases after training (Figure 4B), respectively.

### 3.5. Reproducibility of Is Assessment

**Figure 5:**
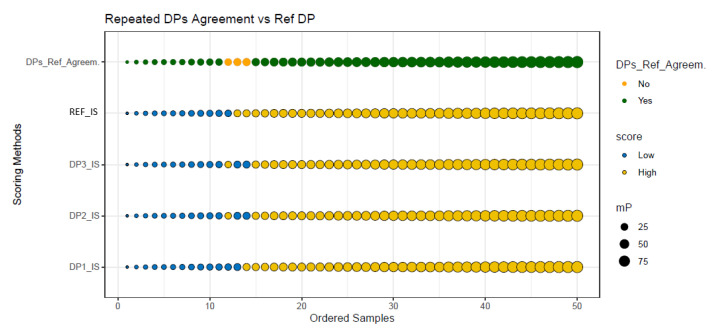
Graphical plot representing the agreement between three repeated IS analyses and reference IS assessment for the 2-category classification. The IS scores for 50 colon cancer cases (x axis) are plotted against the scoring method (horizontal lines, from the bottom to the top, represent three repeated IS analyses [DP1_IS, DP2_IS, and DP3_IS], the reference IS) used (y axis). The mean percentiles (mP) of the CD3+ and CD8+ T-cell densities are represented as circles, whose size is proportional to the value observed for each case. The cases range from the lowest mP to the highest mP and are translated into IS with the 2-category classification: Low IS (mP ≤ 25%) and High IS (mP ˃ 25%). Abbreviations: mP, mean percentile; IS, Immunoscore^®^; DP_IS, digital pathology Immunoscore^®^.

The agreement between three repeated IS scores and the initial reference IS score for each of the 50 CC cases is illustrated in Figure 5. Almost perfect agreement was observed (Cohen’s Kappa of 0.93). In the first repeat (DP1, Figure 5), only 1 out of 50 cases were incorrectly classified as compared to the reference IS result and only two more cases were misclassified in the two remaining repeats (DP2 and DP3, Figure 5), leading to an agreement of 94%. The three discordant cases were very close to the cutoff point of 25% with IS mPs ranging from 21.2% to 26.2%. Thus, IS yielded a sensitivity of 95% and a positive predictive value of 97% (overall agreement of 94%).

## 4. Discussion

The reproducibility of IS digital pathology was previously assessed [2]. Representative images from five centers (Belgium, Canada, China, France, USA) of tissue stained for CD3+ and CD8+ (*n* = 36), having IS ranging from lowest to highest (2.5th to 90th percentiles, respectively) were re-analyzed by eight pathologists from different centers. These eight IS digital pathology quantifications revealed a strong reproducibility (mean cell densities in each tumor region, r = 0.97 for tumor; r = 0.97 for invasive margin; *p* < 0.0001). Only 2.1% variation in the mean percentile of CD3+ and CD8+ T-cell densities was found between IS quantifications. These observations were confirmed in an independent study [20]. This showed the strong reproducibility of IS using digital pathology.

Since visual evaluation of tumor infiltrating lymphocytes in H&E-stained slides by pathologists was not sufficiently accurate for clinical decisions and, as it was important to assess the added value of automated digital pathology over visual assessment on the same CD3+ and CD8+ stains, we evaluated the reproducibility of a visual examination on these slides (T-score) by expert pathologists. The inter-pathologists’ reproducibility and the differences between T-score and automated digital IS were evaluated.

The IS method was confirmed to be a very robust method that produced reliable and consistent data with a very high degree of agreement (94%) between repeated measures. Moreover, the rare cases of discordance (3 out of 50) were all very close to the cutoff value of 25% and re-testing such samples to correctly assign their score would be simple. In contrast, a significant disagreement was observed for the visual semi-quantitative pathologist T-score (High or Low). This inter-observer disagreement was not improved by providing pathologists with training for the visual scoring process to recognize the IS cutoff points of prognostic importance. Furthermore, the study revealed that the effect of training was heterogeneous between pathologists and that, overall, training only marginally improved and, in fact, for two pathologists, worsened the concordance between the visual assessment and IS. Importantly, a high rate of disagreement was observed when comparing the pathologists’ visual assessment with the reference IS, leading to misclassification of almost half the cases (48%) and this disagreement was particularly high for the cases around the 25% clinical cutoff (80%).

The lack of improvement in agreement between pathologist evaluation and quantitative digital pathology, before and after training, is likely multifactorial. In fact, the size of a colon tumor is quite large, and a whole slide analysis revealed a heterogeneous pattern of CD3+ and CD8+ within different areas of the tumor. Furthermore, the mean density of these cells is higher at the invasive margin compared to the core of the tumor, rendering the overall visual evaluation difficult. In addition, these immune cells can be present within the tumor glands or within the stroma at different densities and can be clustered or dispersed even within the same tumor. CD3, encompassing both CD8 and CD4 T-helper cells and CD8 cells, also have different densities in different areas of the tumor, and the evaluation has to be done twice for each of these markers on consecutive slides. Looking at the overall slide is tedious, and the semi-quantitative evaluation of so many heterogeneities is very complex and in fact very subjective. For such evaluation, the novel tool of quantitative digital pathology is clearly much more appropriate, as demonstrated by the poor performance of pathologist scoring, even after training.

**Figure 6:**
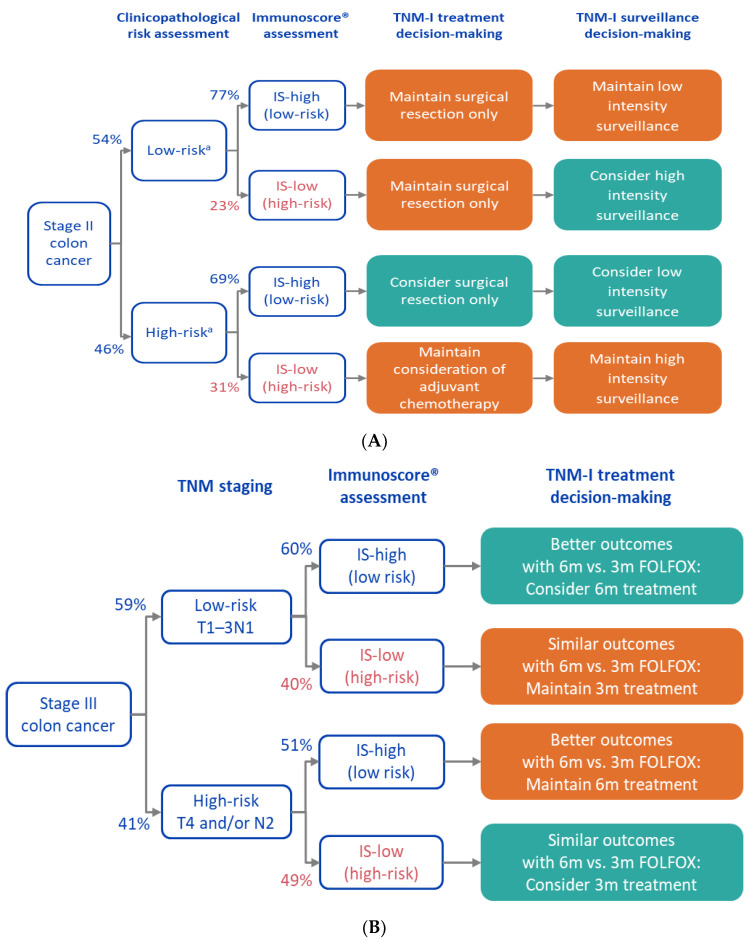
Decision tree for patients with stage II (**A**) and III (**B**) colon cancer considering the IS-High and Low scoring. Abbreviations: 6m, 6 months; 3m, 3 months.

To illustrate how an incorrect determination of an immune response of stage II and III CC patients could influence the subsequent treatment and potential outcome of the patient, we illustrated a clinical decision tree for these patients (Figure 6) [29]. For patients with stage II CC, the misclassification of patients with IS-Low to highly infiltrated tumors (IS-High) results in patients being identified as stage II CC at low clinical risk when they are in fact at high biological risk. This is important because such a situation would produce false expectations of a low risk of recurrence for these patients who will not be monitored as closely as those at high risk of recurrence to detect signs of relapse earlier. Based on the worst-case negative agreement between visual T-score and IS observed in this study (25%), 75% of IS-Low CC cases would be classified as having a good outcome. Thus, 17% of low-risk stage II or 9% of all stage II patients would not be appropriately considered as high-risk patients. They may be undertreated and under screened. Conversely, misclassification of truly IS-High stage II CC patients as having tumors with low immune cell infiltration could result in patients recommended for adjuvant chemotherapy when their recurrence risk is low, and thus they are unnecessarily exposed to long-term toxicity and side-effects of chemotherapy (Figure 6). In the worst-case scenario observed in this study (positive agreement of 79%), this represents 7% of all stage II CC patients who might be overtreated.

In the case of stage III, if IS-Low CC patients were misclassified as IS-High, they would not be identified as poor responders and may be unnecessarily subjected to additional therapy (six months versus three months) and its associated long-term toxicity and intense side effects (Figure 6). Considering the worst incorrect classification observed in this study, 75% of the IS-Low patients would be identified as good responders to extended adjuvant treatment, which represents 37% in the stage III high clinical risk group (T4/N2) or 15% of all stage III CC patients.

Finally, in the worst-case scenario observed, up to 21% of IS-High cases might be incorrectly identified as poor responders to six months of chemotherapy (IS Low) and thus be subjected to an increased risk of relapse.

Altogether, given an estimated 101,420 and 23,000 new stage II and new stage III CC patients per year, respectively, pathologist visual evaluation of T-score would lead to 8700/5800 (before/after training) CC cases being misclassified annually and possibly receiving inappropriate patient care.

A limitation of the study relates to the sample size and to the relatively low number of expert pathologists who evaluated the CD3+- and CD8+-stained images. These results should be validated with a larger cohort of patients and with a larger number of expert pathologists. However, given the very important difference between pathologist T-score classification and the reproducible IS quantification, these results confirm the importance of new tools for pathologists, namely quantitative digital pathology.

## 5. Conclusions

The potential negative impact that misclassification of immune response assessment and thus erroneous prognosis and risk evaluation might have on the clinical management of patients with CC was shown to be significant.

Our results showed that the IS assay provided the best stratification of patients into prognostic recurrence groups (low versus high). We conclude that the standardized and robust IS assay outperforms the assessment of expert pathologists in the clinical setting for immune response evaluation and can thus provide the most appropriate individualized therapeutic decisions for patients with CC.

## Figures and Tables

**Table 1:** Pathologist IS training session steps.

Training Session	Density Value (Mean Percentile)	Reference Images Description	Nr of Images
(1) Cutoff point recognition	25%	CD3+ in the CT and IM region display 25% density	1
		CD8 +in the CT and IM region display 25% density	1
	70%	CD3+ in the CT and IM region display 70% density	1
		CD8+ in the CT and IM region display 70% density	1
(2) A single Immunoscore^®^ may reflect heterogeneity of densities	25%	The IM and CT display similar densities (CD3+ and CD8+)	2
		The IM is more invaded than the CT region (CD3+ and CD8+)	2
		The CT is more invaded than the IM region (CD3+ and CD8+)	2
	70%	The IM and CT display similar densities (CD3+ and CD8+)	2
		The IM is more invaded than the CT region (CD3+ and CD8+)	2
		The CT is more invaded than the IM region (CD3+ and CD8+)	2

## Data Availability

Authors agree to make data and materials supporting the results or analyses presented in their paper available upon reasonable request.
